# A genomic sequence of the type II-A clustered regularly interspaced short palindromic repeats (CRISPR)/CRISPR-associated system in *Mycoplasma salivarium* strain ATCC 29803

**DOI:** 10.1080/20002297.2021.2008153

**Published:** 2022-01-02

**Authors:** Harumi Mizuki, Yu Shimoyama, Taichi Ishikawa, Minoru Sasaki

**Affiliations:** Division of Molecular Microbiology, Department of Microbiology, Iwate Medical University, Shiwa-gun, Japan

**Keywords:** Clustered regularly interspaced short palindromic repeats, CRISPR/Cas system, CRISPR RNA-guided endonuclease, Cas9, *Mycoplasma*, *Mycoplasma salivarium*, *de novo* genome sequencing

## Abstract

**Introduction:**

Clustered regularly interspaced short palindromic repeats (CRISPR)/CRISPR-associated systems are RNA-mediated adaptive immune systems that actagainst invading genetic elements such as phages or plasmids. CRISPR/Cas systems exist in nearly half of bacteria. *Mycoplasma salivarium* is a commensal species of the oropharynx. The American Type Culture Collection maintains five *M. salivarium* strains: ATCC 14277, 23064, 23557, 29803, and 33130. The genome sequence of ATCC 23064 revealed that it has an incomplete CRISPR/Cas system. However, the genome sequences of the remaining strains have not been analyzed.

**Methods:**

We performed polymerase chain reaction-amplicon sequencing and *de novo* genome sequencing to evaluate the presence of the CRISPR/Cas system in four strains.

**Results:**

Only ATCC 29803 possessed *cas1, cas2, cas9*, and *csn2* genes, a CRISPR array, and tracrRNA. The sequences of most components were identical between the CRISPR/Cas systems of ATCC 29803 and ATCC 23064, whereas the spacer sequences and a region of the *cas9* gene were different. Unlike the CRISPR/Cas system of ATCC 23064, the *cas9* gene of ATCC 29803 was not disrupted by the presence of stop codons.

**Conclusion:**

ATCC 29803 possesses genomic components required to express the type II-A CRISPR/Cas system, which potentially functions as an RNA-guided endonuclease.

## Introduction

The clustered regularly interspaced short palindromic repeats (CRISPR)/ CRISPR associated (Cas) system is an RNA-mediated adaptive immune system that acts against invading genetic elements, such as phages or plasmids. The CRISPR/Cas defense system operates in three phases: 1) adaptation: new spacers are acquired from invading nucleic acids and integrated into the CRISPR array; 2) expression: the CRISPR array is transcribed and processed into small interfering CRISPR RNA (crRNA); 3) interference: crRNAs guide the Cas machinery to specifically cleave homologous invasive nucleic acids [1].

This system consists of a CRISPR array and *cas* genes [2]. The CRISPR array contains a leader, direct repeats (DRs), and spacers. DRs are highly conserved and tandem short DNA repeats, whereas spacers are unique sequences located at regular intervals between the DRs. Many of these spacers have been matched to sequences, which originate from extra-chromosomal sources, such as phages, plasmids, or other transferable elements, and are termed protospacers. The *cas* genes encode Cas proteins, which provide the enzymatic machinery required for the acquisition of new spacers from invading genetic elements and for targeting them.

The CRISPR/Cas systems have been reported in 46% of the bacterial species and 82% of the archaea studied [3]. The oral microbiome contains approximately 700 prokaryote species harboring known CRISPR/Cas systems and unidentified CRISPR/Cas systems [4]. Although this system has been classified into six types (type I–VI) based on the signature protein families and features of the *cas* gene loci architecture [5], oral bacterial CRISPR/Cas systems fall mainly under types I and II [6]. Type I systems contain the signature gene *cas3* (or variants of the *cas3* gene), while type-II systems are characterized by the presence of *cas9, cas1*, and *cas2* genes [7]. The type-II CRISPR/Cas system has been further classified into three subtypes: A, B, and C. Type II-A systems comprise of the *cas1, cas2*, and *cas9* genes, as well as the *csn2* gene, which is a signature gene of this subtype [7]. In contrast, type II-B comprises of the *cas4* gene in place of *csn2* gene, while type II-C has only three *cas* genes, *cas1, cas2*, and *cas9* [7].

*Mycoplasma* spp. are the smallest free-living bacteria capable of self-replication and are characterized by the lack of a bacterial cell wall [8]. At present, 16 species of *Mycoplasma* have been identified that infect humans. Among them, *Mycoplasma pneumoniae, M. salivarium, M. buccale, M. orale, M. faucium, M. lipophilum*, and *M. fermentans* are commensal organisms in the oropharynx [9]. *M. salivarium* and *M. orale* are isolated more frequently from the oropharynx than the other *Mycoplasma* spp [9].

A global survey performed to identify and compare the CRISPR/Cas systems in the genome of Mollicutes reported that complete or degraded systems were found in the genome of 13 out of 35 *Mycoplasma* spp [10]. All of these 13 *Mycoplasma* spp. (*M. salivarium, M. lipofaciens, M. synoviae, M. cynos, M. canis, M. dispar, M. ovipneumoniae, M. hyosynoviae, M. arginini, M. spumans, M. arthritidis, M. mobile*, and *M. gallisepticum*) possessed complete or degraded type II-A CRISPR/Cas systems.

The genome size of *Mycoplasma* spp. is extremely small, ranging from 580 to 1,360 kbp, and the number of protein-coding sequences in the genomes is also small, ranging between 475 and 1,545 in 38 strains from 22 spp. studied [11]. Most *Mycoplasma* spp. are considered to be parasites and certain *Mycoplasma* spp. have developed mechanisms to enter host cells that are not naturally phagocytic [8]. Immunohistochemistry and immunoelectron microscopy experiments have shown that *M. salivarium* often colonizes in the epithelial cells of oral leukoplakia [12,13]. Oral leukoplakia is an oral potentially malignant disorder characterized by various site-specific chromosomal abnormalities and gene alterations in epithelial cells [14–17]; however, its underlying mechanism has not yet been clarified. The relationship between *M. salivarium* in epithelial cells and the development and/or progression of oral leukoplakia is also unknown.

In the immune defense mechanism of the type-II CRISPR/Cas systems, the components of the system are first transcribed into trans-activating crRNA (tracrRNA), Cas9 protein, and pre-crRNA. Subsequently, the pre-crRNA is cleaved into crRNA at the site of the DRs by ribonuclease III. Finally, the crRNA-tracrRNA-Cas9 complex forms an active crRNA-guided endonuclease, and the crRNA-targeting sequence binds to a target genomic sequence via complementary RNA-DNA base pairing, after which the DNA is cleaved to form double-strand breaks (DSBs) at the binding site [1]. There has been increasing evidence suggesting that the CRISPR/Cas systems in the human oral microbiome have not only a canonical immune defense mechanism, but also several alternative mechanisms, which can affect bacterial physiological processes such as DNA repair, acquisition of resistance genes, regulation of interspecific competition and intraspecific diversification, and gene expression regulation [6].

Some *Mycoplasma* spp. are reported to fuse with the host cells under appropriate conditions, and during the fusion process, the functioning of the host cells is affected [8]. *Mycoplasma* nucleases, which may degrade host cell DNAs, were presumed to be the crucial factor facilitating this process [8]. Electron microscopic observation of *M. salivarium* cells in epithelial cells signified a fusion between the mycoplasma cells and the cytoplasm of the host cells, and the cell membranes of the mycoplasma cells were not observed in the images [13]. There is a possibility that intracellularly localized *M. salivarium* affects the host epithelial cells through mechanisms involving the CRISPR/Cas system. Therefore, investigating the CRISPR/Cas system of *M. salivarium* is worthwhile.

The American Type Culture Collection (ATCC; Manassas, VA, USA) maintains five strains of *M. salivarium* (ATCC 14277, 23064, 23557, 29803, and 33130) (Table 1). While the complete genome sequence of ATCC 23064 (NCTC 10113) (accession; NCBI: NZ_LR214939) is available on the NCBI database, the genome sequences of the remaining four ATCC strains have not been analyzed previously. The genome sequence of ATCC 23064 includes genes pertaining to the type-II CRISPR/Cas system, namely the CRISPR array, *cas1, cas2*, and *cas9* genes. A frameshift mutation generates a UAA stop codon in the middle of the *cas9* gene sequence, disrupting the *cas9* gene. This frame-shift mutation may impair the crRNA-guided endonuclease function of cas9.

**Table 1.** Description of *Mycoplasma salivarium* strains used in this study

Strain	Other designations	Note
ATCC 14277	Buccal 1	
ATCC 23064	NCTC 10113 NBRC 14478PG 20, H110	type strain
ATCC 23557	Manire A-2-B-3	
ATCC 29803	W	
ATCC 33130	S9	

The CRISPR/Cas systems show considerable variation within species and are not present in all strains of a species. However, when they are present, the number of DRs and spacers varies among the strains in a species [10,18–21]. According to the CRISPR database (CRISPRdb) (https://crispr.i2bc.paris-saclay.fr/crispr/), among the 12 strains of *M. gallisepticum* studied, the number of spacers in the CRISPRs varies between 23 and 105, and the *M. canis* strain PG 14 possesses a CRISPR containing 7 spacers, whereas strain LV contains two CRISPRs comprising 16 and 18 spacers.

It is not known whether the *M. salivarium* strains ATCC 14277, 23557, 29803, and 33130 possess a CRISPR/Cas system. In this study, we explored these four *M. salivarium* strains for the presence of the CRISPR/Cas system and investigated the genomic sequences of the CRISPR/Cas systems in these strains.

## Materials and Methods

### Culture conditions for M. salivarium strains

Five strains of *M. salivarium* (ATCC 14277, 23064, 23557, 29803, and 33130) were purchased from the ATCC (Table 1). ATCC 23064 was used as a positive control for polymerase chain reaction (PCR) amplification and sequencing.

*Mycoplasma* cells were cultured in ATCC medium 243, which was prepared as described by the ATCC. Heart Infusion Broth (17.5 g; Becton-Dickinson, Franklin Lakes, NJ) was dissolved in deionized water (700 mL) and autoclaved at 121°C for 15 min. Heat-inactivated, sterilized horse serum (200 mL; Merck, Darmstadt, Germany) and yeast extract (100 mL; Oriental Yeast, Tokyo, Japan) were added to the solution aseptically.

Each strain was cultured in the medium at 37°C for 7–10 d under anaerobic conditions. The cells were collected via centrifugation at 11,000 × *g* for 20 min, washed twice using Dulbecco’s phosphate-buffered saline without Ca and Mg (D-PBS; Nacalai Tesque, Kyoto, Japan), and resuspended in D-PBS. *Mycoplasma* cells were stored in D-PBS at −20°C.

### Extraction of genomic DNA from M. salivarium strains

Cells suspended in D-PBS were pelleted via centrifugation at 11,000 × *g* for 20 min. Genomic DNA was extracted using the Wizard® Genome DNA Purification Kit (Promega, Fitchburg, WI) according to the manufacturer’s instructions. Following extraction, the genomic DNA samples were stored at −20°C.

### Detection of the CRISPR/Cas system via PCR amplification and sequencing

#### Design of the PCR primers

**Figure 1.**
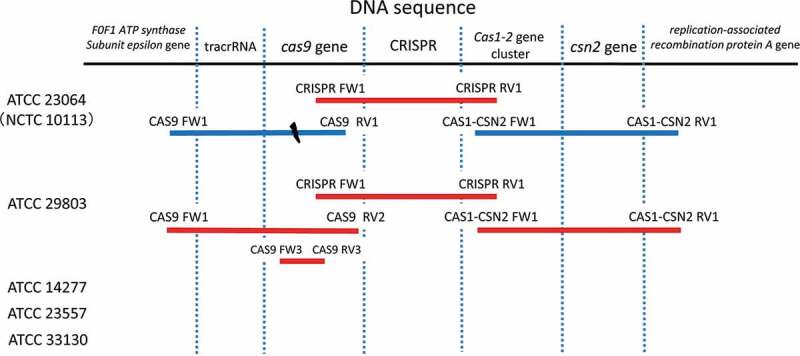
PCR amplification of the CRISPR/Cas system within the genomic sequences of *Mycoplasma salivarium* strains. For ATCC 23064 and ATCC 29803, the genomic sequences were amplified using primer pairs, which are shown at both ends of the bars. For ATCC 14277, 23557, and 33130, the genomic sequences were not amplified using any primer pairs. Red bars indicate that amplified PCR products were sequenced via primer walking or capillary sequencing. Blue bars indicate PCR amplification alone without sequencing. The black dash indicates the disruption in the gene sequence. PCR, polymerase chain reaction; ATCC, American Type Culture Collection; CRISPR, clustered regularly interspaced palindromic repeats; Cas, CRISPR-associated.

To amplify the DNA sequences of the CRISPR/Cas system components(, which comprise the CRISPR array, *csn2, cas1, cas2*, and *cas9*, and the DNA sequence encoding tracrRNA, several PCR primers (Table 2, Figure 1) were designed based on the complete genome sequence of ATCC 23064 (NCTC 10113) using Primer3Plus software (www.bioinformatics.nl/cgi-bin/primer3plus/primer3plus.cgi). The primer CAS9 RV2 (Table 2) was designed based on the flanking sequence of the CRISPR array sequence of ATCC 29803, which was slightly different from that of ATCC 23064. The primers were synthesized by Fasmac Co., Ltd. (Atsugi, Kanagawa, Japan).

**Table 2.** Sequences of the primers used for PCR amplification in this study

Name	Sequence (5′→3′)
CRISPR FW1	AGGTAGTTGTGTTTGATCCCACT
CRISPR RV1	TTTTGCTGCATGCCCTTCAC
CRISPR FW2	TGGCGAGAATCCGAAACTTA
CRISPR RV2	TCGCG GTTAAATTTGCTACC
CRISPR FW3	GGTAAGGTAGTTGTGTTTGATCCCAC
CRISPR RV3	GCATGCCCTTCACGGTTAGA
CAS9 FW1	CTTTCAAAACCACCGAAGGA
CAS9 RV1	AGTTTCGGATTCTCGCCAAA
CAS9 RV2	CTGCGGCTTGTATATGTTTCCC
CAS9 FW3	AGTTTTGGCGGAATTTGGTA
CAS9 RV3	CTTTTCACGTGCCAATTCAA
CAS1-CSN2 FW1	CGCAAATTGTACCATTCAATGG
CAS1-CSN2 RV1	TGCTACTCTGACATCGCCAT
RNC FW	TGTCCATCCACAATAACGCT
RNC RV	AGAGGGGATTGCAACTAAACA

#### PCR amplification

The target region sequence was amplified using the KOD One Master Mix (Toyobo, Osaka, Japan). Deionized water (15 µL), 10 mM forward primer (4.5 µL), 10 mM reverse primer (4.5 µL), and approximately 5 ng/µL of genomic DNA (1 µL) were added to the Master Mix (25 µL) for each tube. Amplification was performed using the following thermocycling program: initial template denaturation at 94°C for 2 min; 30 cycles of denaturation, annealing, and extension; and a final elongation step at 68°C for 6 min. The annealing temperature varied according to the melting temperature of the primers and the manufacturer’s instructions for the polymerase used. The duration of the denaturation, annealing, and extension processes was determined according to the manufacturer’s instructions for the polymerase used.

#### Sequencing via primer walking

The PCR products were analyzed via electrophoresis on 1% agarose gels. PCR products were purified from the gel slices using NucleoSpin® Gel and PCR Clean-up Kits (Takara Bio Inc., Kusatsu, Japan), according to the manufacturer’s instructions. Following extraction, the DNA samples were stored at 0°C until analysis. The PCR products were sequenced via primer walking with paired-end reading, based on segmenting the sequence into several consecutive short sequences [22]. Sequencing was performed by Hokkaido System Science Co., Ltd. (Sapporo, Hokkaido, Japan), using an ABI 3730xl DNA analyzer (Thermo Fisher Scientific, Waltham, MA).

### Detection of the CRISPR/Cas system via de novo genome sequencing

In strains in which PCR could not amplify the DNA sequences of the CRISPR/Cas systems, the CRISPR/Cas systems were analyzed via *de novo* genome sequencing. For short-read sequencing, the MGEasy FS PCR^Free DNA Library Prep set (MGI Tech, Shenzhen, Guangdong, China) was used for library preparation. Subsequently, 2 × 150 bp paired-end sequencing was performed using a DNBSEQ-G400RS FAST sequencing instrument (MGI Tech) according to the manufacturer’s instructions. Raw sequencing data were processed using the FASTQ processing program fastp v. 0.20.1 for trimming low-quality data [23]. Quality-filtered reads were assembled using Unicycler v. 0.4.8. Genome assemblies were annotated automatically using the DNA Databank of DDBJ Fast Annotation and Submission Tool (https://dfast.nig.ac.jp/analysis/annotation).

Comparison of multiple genomic regions was visualized using the genome comparison visualizer Easyfig (Easyfig-home (mjsull.github.io)). Sequences with high similarity to the CRISPR array and the *csn2, cas1, cas2*, or *cas9* genes of ATCC 23064 were searched for in the *de novo* genome assemblies using Basic Local Alignment Search Tool (BLAST). The online CRISPRfinder program (https://crispr.i2bc.paris-saclay.fr/Server/) was used to identify CRISPR arrays in the *de novo* genome assemblies. Since the CRISPR/Cas system is located between the replication-associated recombination protein A (*rarA*) gene and the F0F1 ATP synthase subunit epsilon (*ATP5F1E*) gene in ATCC 23064, these genes were also analyzed using BLAST to help identify the CRISPR/Cas sequences in the *de novo* genome sequences. The *de novo* sequencing and analysis of the genome sequences were performed by the Taniguchi Dental Clinic - Oral Microbiome Center (Takamatsu, Kagawa, Japan).

### Comparison of the CRISPR array and cas gene sequences among the strains

The similarity between the CRISPR array and *cas* gene sequences was analyzed using BLAST followed by Clustal Omega (https://www.ebi.ac.uk/Tools/msa/clustao/).

### Detection of spacers and protospacers

We sought DRs and spacers in the CRISPR using CRISPRfinder and searched for homology to spacers in the genome sequences of phages, plasmids, and viruses to identify the protospacers and their flanking sequences in the databases of Genbank-Phage, RefSeq-Plasmid, and IMG/VR, respectively, using CRISPRTarget [http://crispr.otago.ac.nz/CRISPRTarget/crispr_analysis.html). Homology to spacers in the genomic sequences of bacteria was identified using BLAST against the NCBI database. The protospacers were defined as sequences with homology that had a maximum of two mismatches out of 30 nucleotides, based on the recommendations of 19.

### Prediction of the Cas9 protein sequence

Based on the *cas9* gene sequences, the Cas9 protein sequences were predicted using the ExPASy-Translate tool (https://web.expasy.org/cgi-bin/translate/dna2aa.cgi] by using the genetic code of *Mycoplasma*. The UGA codon serves as a stop codon in most organisms; however, it is translated to tryptophan in *Mycoplasma* spp [24]. The theoretical molecular weight of the Cas9 protein was computed using ExPASy-Compute pI/Mw (https://web.expasy.org/compute_pi/). Based on the predicted Cas9 protein sequence, the Cas9 protein structure was predicted using SOSUI (http://harrier. nagahama-i-bio.ac.jp/sosui/mobile/).

### Detection and prediction of the tracrRNA sequence

The DNA sequence encoding tracrRNA is generally located near the *cas* genes and contains a stretch with almost perfect homology to a CRISPR DR sequence [25]. We analyzed the regions near the *cas* genes using BLAST. The RNA sequences transcribed from the DRs and tracrRNA-coding DNA sequences were concatenated to predict the tracrRNA sequence. The secondary structure of the crRNA/tracrRNA hybrid, which was produced from the concatenated RNA sequence, was simulated using the mfold software (http://www.unafold.org/mfold/applications/rna-folding-form.php). The tracrRNA sequence was determined as follows: the region of the sequence bound to the crRNA was estimated to be 21 nucleotides in length, and the length of the tail region was determined based on the secondary structure of a crRNA/tracrRNA hybrid present in *M. gallisepticum* S6 [10].

### Detection of the rnc gene

To identify the *rnc* genes encoding the RNase III protein, a PCR primer pair, RNC FW and RNC RV (Table 2), was designed with reference to the genomic sequence of ATCC 23064. The *rnc* gene sequences were amplified via PCR, and the products were sequenced. The *rnc* genes were analyzed in the *de novo* genome sequences.

## Results

### Identification of the CRISPR/Cas system via PCR amplification and primer walking, or de novo genome sequencing

**Figure 2.**
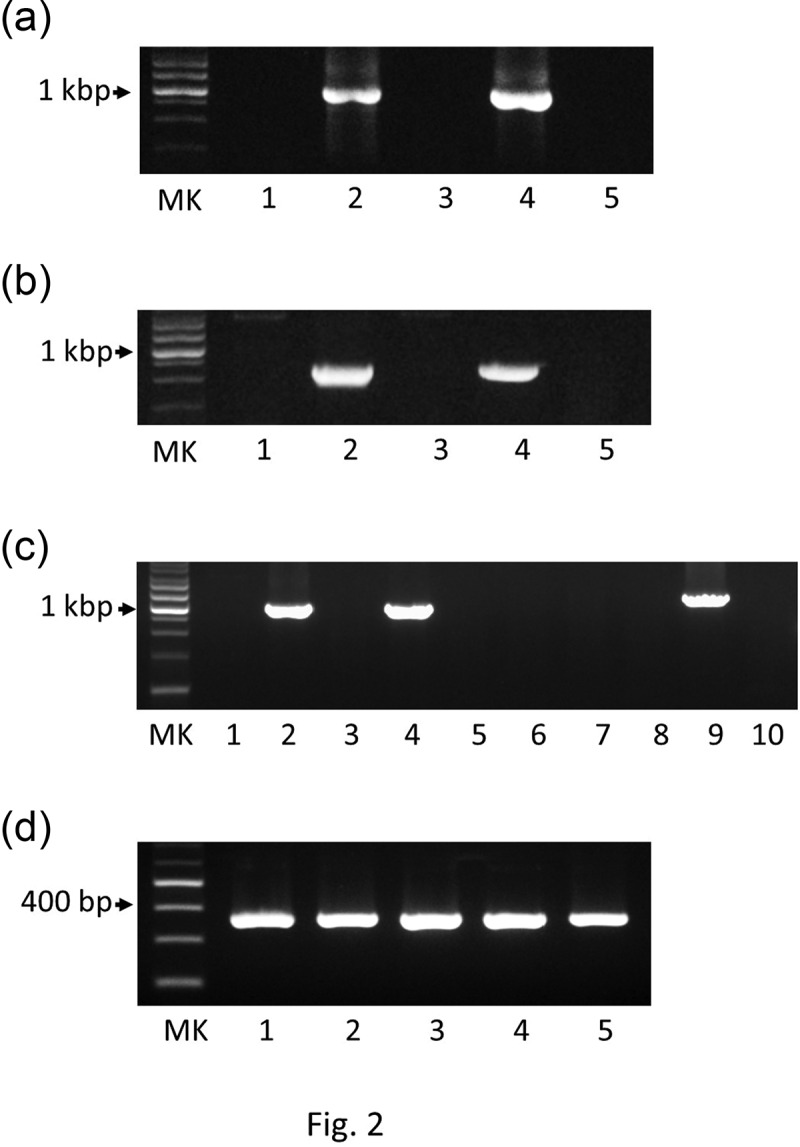
Agarose gel electrophoresis analysis of amplified PCR products. **(a)** Following amplification, the PCR products of the CRISPR sequences of five *M. salivarium* strains were analyzed via agarose gel electrophoresis: ATCC 14277 (lane 1), ATCC 23064 (lane 2), ATCC 23557 (lane 3), ATCC 29803 (lane 4), and ATCC 33130 (lane 5). MK: 1 kbp DNA marker. **(b)** PCR products generated by amplifying the *cas1, cas2*, and *csn2* gene sequences of ATCC 14277 (lane 1), ATCC 23064 (lane 2), ATCC 23557 (lane 3), ATCC 29803 (lane 4), and ATCC 33130 (lane 5). MK: 1 kbp DNA marker. **(c)** PCR amplification of the *cas9* gene regions of five strains using the primer pair CAS9 FW1 and CAS9 RV1 (lanes 1–5) or the primer pair CAS9 FW1 and CAS9 RV2 (lanes 6–10). The template DNA used for PCR included genomic DNA from ATCC 14277 (lanes 1 and 6), ATCC 23064 (lanes 2 and 7), ATCC 23557 (lanes 3 and 8), ATCC 29803 (lanes 4 and 9), and ATCC 33130 (lanes 5 and 10). MK: 1 kbp DNA marker. **(d)** PCR products generated by amplifying the *rnc* gene sequences of five strains: ATCC 14277 (lane 1), ATCC 23064 (lane 2), ATCC 23557 (lane 3), ATCC 29803 (lane 4), and ATCC 33130 (lane 5). MK: 100 bp DNA marker. PCR, polymerase chain reaction; ATCC, American Type Culture Collection; CRISPR, clustered regularly interspaced palindromic repeats.

**Figure 3.**
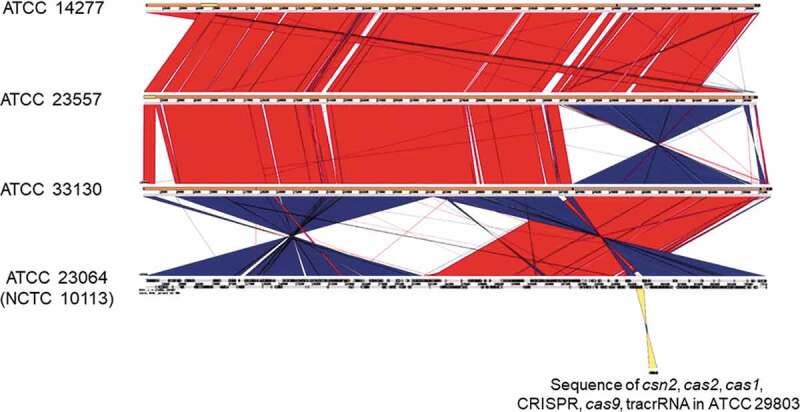
Comparison between the whole genomes of *M. salivarium* ATCC 14277, ATCC 23557, ATCC 33130, and ATCC 23064 (NCTC 10113) strains, visualized using Easyfig. Based on BLAST analysis (red for matches in the same direction and blue for inverted matches), vertical blocks between the sequences indicate regions of shared similarity. Comparison between the whole genome sequence of ATCC 23064 and the sequences of the CRISPR/Cas system in ATCC 29803 is shown as a yellow block. ATCC, American Type Culture Collection; BLAST, basic local alignment search tool; CRISPR, clustered regularly interspaced palindromic repeats; Cas, CRISPR-associated.

The DNA sequences of the CRISPR array and the *csn2, cas1, cas2*, and *cas9* gene loci were amplified via PCR for the strains ATCC 23064 and ATCC 29803 (Figures 1 and 2a–c). Subsequently, the sequences of the CRISPR/Cas system were analyzed via primer walking using PCR products. The CRISPR/Cas system sequences of the strains ATCC 14277, 23557, and 33130 were not amplified using any of the primer pairs shown in Table 2 (Figures 1 and 2a–c), or the other primers. Therefore, *de novo* genome sequencing was performed for these strains. A summary of the *de novo* genome assembly of these strains is shown in Table 3. The total genome sequence lengths of ATCC 14277 (accession; DDBJ: BPLV01000001–01000007) and ATCC 23557 (accession; DDBJ: BPLW01000001–01000006) were almost the same, and that of ATCC 33130 (accession; DDBJ: BPLX01000001–01000009) was slightly longer (Table 3). However, the genome size was similar to that of the complete genome sequence of ATCC 23064. Comparison of multiple genomic sequences using BLAST showed high similarity among the three strains and ATCC 23064, as visualized using Easyfig (Figure 3). Although the *rarA* and *ATP5F1E* genes were identified in the *de novo* genome assemblies of three strains with high similarity to that of ATCC 23064, neither the whole, nor partial sequences, of the CRISPR array, *csn2, cas1, cas2*, or *cas9* genes were found in the region between the *rarA* gene and the *ATP5F1E* gene, or elsewhere (Figure 3). A CRISPR/Cas system was not found in ATCC 14277, 23557, and 33130.

**Table 3.** Summary of the *de novo* genome assembly for *Mycoplasma salivarium* strains ATCC 14277, 23557 and 33130

Element	ATCC 14277	ATCC 23557	ATCC 33130	ATCC 23064 (NCTC 10113) *
Total sequence length (bp)	718,941	718,986	736,914	728,347
Number of sequences	7	6	9	1
Longest sequences (bp)	551,285	702,908	503,089	
N50 (bp)	551,285	702,908	503,089	
Gap ration (%)	0.0	0.0	0.0	
GC content (%)	26.5	26.5	26.5	
Number of CDSs	610	618	627	614
Average protein length	142.3	142.7	140.6	
Coding ratio (%)	91.7	90.6	90.7	
Number of rRNAs	3	3	3	3
Number of tRNAs	33	33	33	33
Number of CRISPRs	0	0	0	1

*Data from NCBI Reference Sequence: NZ_LR214938.2

### Identification of the CRISPR sequence in ATCC 29803

The sequences of the CRISPR regions that were amplified using the primer pair CRISPR FW1 and CRISPR RV1 were analyzed via primer walking for ATCC 23064 and ATCC 29803. In ATCC 23064, sequencing of the CRISPR region was performed to confirm PCR amplification and sequencing accuracy. The determined sequence of the CRISPR array of ATCC 23064 (accession; DDBJ: LC633536) was identical to that in the genome sequence of ATCC 23064 available on the NCBI database.

In strains ATCC 23064 and ATCC 29803, the sequences contained 28 copies of a 36 bp DR. The DRs were separated by 30 bp unique sequences. These structures corresponded to the CRISPR comprising DR consensus sequences and regularly interspersed spacer sequences.

All the DR consensus sequences were identical in ATCC 23064 (Table 4), whereas certain variants were found in the ATCC 29803 sequence (accession; DDBJ: LC628936). Sixteen DRs had a sequence of 5′-GTTTTAGCGCTGTACAATATTTGAGTAAGCTATAAC-3′, which was identical to that of ATCC 23064; however, eight sequences were 5′-GTTTTAGTGCTGTACAATATTTGAGTAAGCTATAAC-3′, two sequences were 5′-GTTTTAGCGCTGTACAATATTTGAGTAAGTTATAAC-3′, and one sequence was 5′-GTTTTAGCACTGTACAATATTTGAGTAAGCTATAAC-3′. The 26^th^ repetitive sequence (5′-GTTTTAGCGCTGTACAATATTTGA__AAC-3′) was 9 nucleotides (GTAAGCTAT) shorter than the other DR sequences (Table 5).

**Table 4.** Direct repeat consensus sequence and spacer sequence in ATCC 23064

No.	Direct repeat consensus sequence (36 bp)	Spacer sequence (30 bp)
1	GTTTTAGCGCTGTACAATATTTGAGTAAGCTATAAC	TTTCTTCTCCTGCTCCTGTTGGTTTTGCTC
2	GTTTTAGCGCTGTACAATATTTGAGTAAGCTATAAC	TCATTTAATATAAAAAAAACAACAAGGAAA †
3	GTTTTAGCGCTGTACAATATTTGAGTAAGCTATAAC	TCATTTAATATAAAAAAAACAACAAGGAAA †
4	GTTTTAGCGCTGTACAATATTTGAGTAAGCTATAAC	TTACACAAGATATGATTAACAACCCAACAA
5	GTTTTAGCGCTGTACAATATTTGAGTAAGCTATAAC	TTTATAATTACATCACATTCTTGACATATA
6	GTTTTAGCGCTGTACAATATTTGAGTAAGCTATAAC	TTGACGCAAAAATTTATGGTAATATTCCAG
7	GTTTTAGCGCTGTACAATATTTGAGTAAGCTATAAC	GAAGACGTTTTAATATATTCTAAATATTCA ‡
8	GTTTTAGCGCTGTACAATATTTGAGTAAGCTATAAC	TAATTTTGTTGATATTCAATTTAATTTGAT
9	GTTTTAGCGCTGTACAATATTTGAGTAAGCTATAAC	GAAAAAAGGTAGAGTTAGCAGGACTAACAA
10	GTTTTAGCGCTGTACAATATTTGAGTAAGCTATAAC	CTCTCTAAAGAAAATGAATATTTGAGAAGC
11	GTTTTAGCGCTGTACAATATTTGAGTAAGCTATAAC	GCTGAACGTATCATTAGAAAACGTGCAAAA
12	GTTTTAGCGCTGTACAATATTTGAGTAAGCTATAAC	AACACAAGAAAACAACAAAGAATTACAGCT
13	GTTTTAGCGCTGTACAATATTTGAGTAAGCTATAAC	ATTATTGCTTTATTGATTGATATGAAGTAC §
14	GTTTTAGCGCTGTACAATATTTGAGTAAGCTATAAC	TCATTAAAGCAACTTAATAGTTGTGATAAC
15	GTTTTAGCGCTGTACAATATTTGAGTAAGCTATAAC	TTTAATATCTAACTAAGAAAAAGCGAGCAC
16	GTTTTAGCGCTGTACAATATTTGAGTAAGCTATAAC	GTAAACTAATTCTTATAATTTTCCTTTAAG ††
17	GTTTTAGCGCTGTACAATATTTGAGTAAGCTATAAC	TAGAATAAGTATTATCTCAATCATTGTAAT ‡‡
18	GTTTTAGCGCTGTACAATATTTGAGTAAGCTATAAC	GAAGACGTTTTAATATATTCTAAATATTCA ‡
19	GTTTTAGCGCTGTACAATATTTGAGTAAGCTATAAC	ATTATTGCTTTATTGATTGATATGAAGTAC §
20	GTTTTAGCGCTGTACAATATTTGAGTAAGCTATAAC	TCATTAAAGCAACTTAATAGTTGTGATAAC
21	GTTTTAGCGCTGTACAATATTTGAGTAAGCTATAAC	TTGCAGCATTAACATTAACCATTGATGCTA
22	GTTTTAGCGCTGTACAATATTTGAGTAAGCTATAAC	TAGAATAAGTATTATCTCAATCATTGTAAT ‡‡
23	GTTTTAGCGCTGTACAATATTTGAGTAAGCTATAAC	GAAGACGTTTTAATATATTCTAAATATTCA ‡
24	GTTTTAGCGCTGTACAATATTTGAGTAAGCTATAAC	ATTATTGCTTTATTGATTGATATGAAGTAC §
25	GTTTTAGCGCTGTACAATATTTGAGTAAGCTATAAC	TCATTAAAGCAACTTAATAGTTGTGATAAC
26	GTTTTAGCGCTGTACAATATTTGAGTAAGCTATAAC	TACTATAAAATTACCATCTCAACTTAAATT
27	GTTTTAGCGCTGTACAATATTTGAGTAAGCTATAAC	GTAAACTAATTCTTATAATTTTCCTTTAAG ††
28	GTTTTAGCGCTGTACAATATTTGAGTAAGCTATAAC	

Spacer sequences with the symbols † (No. 2, 3), ‡ (7, 18, 23), § (13, 19, 24), ¶ (14, 20, 25), †† (16, 27) and ‡‡ (17, 22) indicate to be identical respectively.

**Table 5.** Direct repeat consensus sequence and spacer sequence in ATCC 29803

No.	Direct repeat consensus sequence (36 bp)	Spacer sequence (30 bp)
1	GTTTTAGCGCTGTACAATATTTGAGTAAGCTATAAC	AGTATAGTGGACGTTAATGCAAACCAAAAA
2	GTTTTAGCGCTGTACAATATTTGAGTAAGCTATAAC	AATTCATAGATGGTTGAACGTATAAAAAAG
3	GTTTTAGCGCTGTACAATATTTGAGTAAGCTATAAC	ATTATTATTGGTCATTTTCACGAAATAGAA
4	GTTTTAGCGCTGTACAATATTTGAGTAAGCTATAAC	TCGGTCCCAGAAACTTGAATAGACAATTAA †
5	GTTTTAGTGCTGTACAATATTTGAGTAAGCTATAAC	TCGGTCCCAGAAACTTGAATAGACAATTAA †
6	GTTTTAGCGCTGTACAATATTTGAGTAAGCTATAAC	GATAATCAAGCAAAAGATTAAGACAATTAC
7	GTTTTAGCGCTGTACAATATTTGAGTAAGCTATAAC	ATCCAACAATTATAAATATAACATCACCAG
8	GTTTTAGCGCTGTACAATATTTGAGTAAGCTATAAC	CAGACCATGCAGTTTCATTATTGTTTGGAC
9	GTTTTAGTGCTGTACAATATTTGAGTAAGCTATAAC	CACCTTTAGGCTATGCACAAGGCTTTAAAA
10	GTTTTAGCGCTGTACAATATTTGAGTAAGCTATAAC	TAACAGTAATTTCAATTATATATGATCTTT
11	GTTTTAGCGCTGTACAATATTTGAGTAAGCTATAAC	ATCTGGAGTACAAAAGATAGCATTAATTTA
12	GTTTTAGCGCTGTACAATATTTGAGTAAGTTATAAC	AAATAAGACTAGAAGAAAGAGAACAAGAGA
13	GTTTTAGCGCTGTACAATATTTGAGTAAGTTATAAC	TATTAGAACTACAAAAACTAAAAGAACACA
14	GTTTTAGCGCTGTACAATATTTGAGTAAGCTATAAC	TTTCTAATTATTTTGCATCCAACTTTACAG
15	GTTTTAGTGCTGTACAATATTTGAGTAAGCTATAAC	TAACATTCATTACACTATTAGATAACTCAA
16	GTTTTAGCGCTGTACAATATTTGAGTAAGCTATAAC	ATTTTGTTCATTATTTAAGATATTTAGATT
17	GTTTTAGTGCTGTACAATATTTGAGTAAGCTATAAC	TCTAAAGCCTGTTTTTATATAAACTTACCT
18	GTTTTAGTGCTGTACAATATTTGAGTAAGCTATAAC	TGTTATTTAGTCATTTTCTATTTGTATATT
19	GTTTTAGCGCTGTACAATATTTGAGTAAGCTATAAC	CATTTACTGGTTTATTGCCTTGTTTAACTA
20	GTTTTAGTGCTGTACAATATTTGAGTAAGCTATAAC	ATTTAATAAAAAATACTTATATTGCGAATA
21	GTTTTAGCACTGTACAATATTTGAGTAAGCTATAAC	AACATTAACCAAATATATATGCAAATACTA ‡
22	GTTTTAGTGCTGTACAATATTTGAGTAAGCTATAAC	TGTAATTGTAGTTATGTTGTCTTCTCATAA §
23	GTTTTAGCGCTGTACAATATTTGAGTAAGCTATAAC	AACATTAACCAAATATATATGCAAATACTA ‡
24	GTTTTAGTGCTGTACAATATTTGAGTAAGCTATAAC	TGTAATTGTAGTTATGTTGTCTTCTCATAA §
25	GTTTTAGCGCTGTACAATATTTGAGTAAGCTATAAC	TTAAAGAATATAAAACGCAAATTCCTAGTT
26	GTTTTAGCGCTGTACAATATTTGAAAC	ACAAGCATAAACAAGAAGTTTTAGAAGTTG
27	GTTTTAGCGCTGTACAATATTTGAGTAAGCTATAAC	TAGCAAAAGCAATTAAAAAACTAAATATTA
28	GTTTTAGCGCTGTACAATATTTGAGTAAGCTATAAC	

Red letters in the direct repeat consensus sequence indicate the mismatches with the direct repeat consensus sequence ‘GTTTTAGCGCTGTACAATATTTGAGTAAGCTATAAC’. Spacer sequences with the symbols † (No. 4, 5), ‡ (21, 23) and § (22, 24) indicate to be identical respectively.

The 30 bp unique sequences were separated regularly by DRs, which corresponded to spacers. Most of the spacer sequences were unique in both strains (Tables 4 and 5); however, six repeated spacers were found in ATCC 23064 (Table 4). In ATCC 29803, three repeated spacers were identified (Table 5).

A part of the flanking sequence of the CRISPR of ATCC 29803 showed 100% similarity to the partial *cas1* gene sequence of ATCC 23064. Moreover, a 127 bp sequence was found that was located between the CRISPR and the *cas1* gene locus, and adjacent to the first DR. This sequence was as follows.TAAAAACTCCTTATATTTAATT. AGCCATAACATGGCTACATATAATTATAAAGCAGTTGTTTTAATGCAAAAATTGACTAAAAATGCATAAAAATAGCGTTTTTTTGACAAAATCAACAAAAATGAG. The sequence was identical to that of ATCC 23064 except for one nucleotide, and it appeared to correspond to the leader sequence, which was located adjacent to the first DR and is usually conserved among species [26]. The leader sequence is generally located upstream of the CRISPR [26]; thus, we placed this sequence on the 5′ side of the CRISPR in this study. Therefore, the DNA sequences of the CRISPR array and *cas* genes of ATCC 29803 were complementary to those in the genome sequence of ATCC 23064 obtained from the NCBI database. The sequences downstream of the CRISPR arrays, the *cas9* gene sequences, and the intergenic spacer sequences between the CRISPR array and the *cas9* gene locus differed between ATCC 23064 and ATCC 29803.

### Identification of the protospacers in the CRISPR of ATCC 29803

For ATCC 29803, spacer homologies with a maximum of two mismatches out of 30 nucleotides were not identified in any phage or plasmid genome sequence. Two spacer sequences, numbers 9 and 12, matched a region of the coding sequences of *M. salivarium* strains with high similarity (Table 6). The ninth spacer matched a sequence in the uracil-DNA glycosylase gene of ATCC 23064 with one mismatch and ATCC 14277 with no mismatch. Although similar sequences were identified for the same genes of ATCC 23557 and ATCC 33130, they matched the ninth spacer with three mismatches. The twelfth spacer was similar to sequences of the L-lactate dehydrogenase (*Ldh*) genes of ATCC 23064, ATCC 14277, ATCC 23557, and ATCC 33130 with one mismatch.

**Table 6.** Potential protospacer sequences matched with the spacer sequence in ATCC 29803

Spacer No.	Spacer sequence	Protospacer match (annotation)	5′ Flankingsequence	Potential protospacer sequence	3′ Flanking sequence
9	CACCTTTAGGCTATGCACAAGGCTTTAAAA	*Mycoplasma salivarium* ATCC 23064 (NCTC10113)(**uracil-DNA glycosylase gene**)	ATATGTGAAT	CTTTAAAACCTTGTGCATAGCCTAAA**G**GTG	AGGGATGAGA
		*Mycoplasma salivarium* ATCC 114277(**uracil-DNA glycosylase gene**)	TCTCATCCCT	CACCTTTAGGCTATGCACAAGGCTTTAAAA	ATTCACATAT
12	AAATAAGACTAGAAGAAAGAGAACAAGAGA	*Mycoplasma salivarium* ATCC 23064 (NCTC10113)(**L-lactate dehydrogenase gene**)	CATTTGCCCA	AAATAAGATTAGAAGAAAGA**G**AACAAGAGA	TGTTTGATAA
		*Mycoplasma salivarium* ATCC 114277(**L-lactate dehydrogenase gene**)	TTATCAAACA	TCTCTTGTTCTCTTTCTTTTAGTCTTATTT	TGGGCAAATG
		*Mycoplasma salivarium* ATCC 23557(**L-lactate dehydrogenase gene**)	TTATCAAACA	TCTCTTGTTCTCTTTCTTTTAGTCTTATTT	TGGGCAAATG
		*Mycoplasma salivarium* ATCC 33130(**L-lactate dehydrogenase gene**)	CATTTGCCCA	AAATAAGATTAGAAGAAAGA**G**AACAAGAGA	TGTTTGATAA

Red letters in the potential protospacer sequence indicate the mismatches with the spacer sequences.

### Identification of the cas gene sequences, and prediction of the Cas9 protein in ATCC 29803

In ATCC 29803, the sequences of the *cas1, cas2*, and *csn2* genes were amplified via PCR (Figures 1 and 2b), and the PCR products were sequenced. The sequences of *cas1, cas2*, and *csn2* showed 100%, 99%, and 99% similarity, respectively, to those of ATCC 23064.

The *cas9* gene sequence of ATCC 23064 was amplified using the primer pair CAS9 FW1 and CAS9 RV1. In ATCC 29803, this sequence was amplified using the primer pairs CAS9 FW1 and CAS9 RV1, and CAS9 FW1 and CAS9 RV2 (Figures 1 and 2c). In ATCC 14277, 23557, and 33130, the *cas9* gene sequences were not amplified via PCR (Figures 1 and 2c).

In ATCC 29803, the sequence of the PCR amplicon generated using the CAS9 FW1 and CAS9 RV2 primer pair was determined. The complete *cas9* gene sequence was then obtained via assembly of this sequence and the flanking sequence of the CRISPR array. A region of the *cas9* gene sequence was also analyzed via PCR amplification using the primer pair CAS9 FW3 and CAS9 RV3 (Table 2), as shown in Figure 1, and capillary sequencing with paired-end reading. The analyzed sequence (480 bp) completely matched the sequence obtained via primer walking (sequence data are not shown).

The *cas9* gene sequences of ATCC 23064 and ATCC 29803 were compared using Clustal Omega (see Supplementary Data 1). Using BLAST, the *cas9* sequences were found to show 98% similarity. The unique portion comprised a region of approximately 500 bp that was adjacent to the CRISPR array. The 500 bp sequence of ATCC 29803 showed no significant similarity to that of ATCC 23064. The *cas9* of ATCC 23064 is disrupted in the middle of the gene sequence by a frameshift mutation generated by a UAA stop codon. The sequence of ATCC 29803 had no stop codon within the *cas9* gene sequence (Supplementary Data 1). Based on the *cas9* gene sequence, the Cas9 protein sequence of ATCC 29803 was predicted to contain 1,203 amino acids (Supplementary Data 2). The theoretical molecular weight was 141.0 kDa, and the protein was predicted to be soluble.

### Identification of the tracrRNA-coding DNA sequence and prediction of the secondary structure of a crRNA/tracrRNA hybrid in ATCC 29803

**Figure 4.**
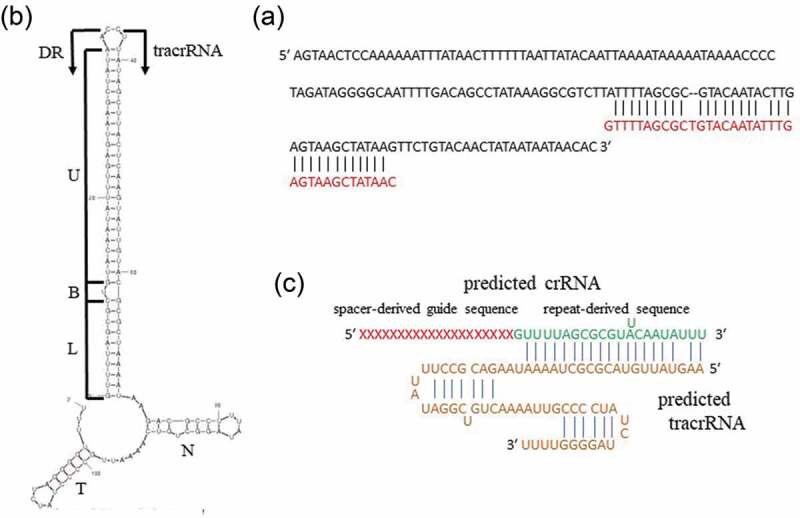
Prediction of the tracrRNA sequence of *M. salivarium* ATCC 29803. **(a)** The tracrRNA-coding sequence contained a 36 bp stretch with 88.9% homology to a DR sequence (red letters). The tracrRNA-coding sequence is included in an array containing a homology part and the 5′ side part. **(b)** Secondary structure of a DR/tracrRNA hybrid, simulated by concatenating the RNA sequences of a DR and the tracrRNA sequence, predicted using mfold. A predicted stem involving a DR and a tracrRNA anti-repeat includes a lower stem (L), a bulge (B), and an upper stem (U). N: nexus stem-loop; T: terminator. The tracrRNA sequence terminated with a poly-uridine tract (UUU), based on the secondary structure of a crRNA/tracrRNA hybrid of *Mycoplasma gallisepticum* S6 as a reference. **(c)** Simulation of binding of a predicted crRNA and a tracrRNA. A repeat-derived sequence at the 3′ end of crRNA was postulated to have 22 nucleotides based on a reference sequence of *Streptococcus pyogenes* crRNA. ATCC, American Type Culture Collection; crRNA, clustered regularly interspaced palindromic repeats-associated RNA; tracrRNA, transactivating crRNA; DR, direct repeat.

A 158 bp DNA sequence encoding tracrRNA (5′-AGTAAGTCCAAAAAATTTATAACTTTTTTAATTATACAATTAAAATAAAAATAAAACCCCTAGATAGGGGCAATTTTGACAGCCTATAAAGGCGTCTTATTTTAGCGCGTACAATACTTGAGTAAGCTATAAGTTCTGTACAACTATAATAATAACAC-3′) that contained a 36 bp stretch with 88.9% (32/36 bp) homology to a DR sequence, was identified downstream of the *cas9* gene locus in ATCC 29803 (Figure 4(a)). This sequence was identical to that of ATCC 23064, except for one nucleotide. The length of the tracrRNA tail was postulated to be 46 nucleotides, and the secondary structure of the DR/tracrRNA hybrid was predicted to consist of a stem involving the DR and tracrRNA anti-repeats, two stem-loops (a nexus stem-loop and a terminator), two linkers, and a poly-uridine tract, similar to the secondary structure of a crRNA/tracrRNA hybrid present in *M. gallisepticum* S6 [10] (Figure 4(b)). Therefore, assuming that the length of the repeat-derived 3′-sequence of crRNA is 22 nucleotides, which is the same length as that of *Streptococcus pyogenes* crRNA [27], the tracrRNA was predicted to be 67 nucleotides in length, with the following sequence: 5′-AAGUAUUGUACGCGCUAAAAUAAGACGCCUUUAUAGGCUGUCAAAAUUGCCCCUAUCUAGGGGUUUU-3′ (Figure 4(c)).

### Arrangement of the CRISPR/Cas system components in ATCC 29803

**Figure 5.**
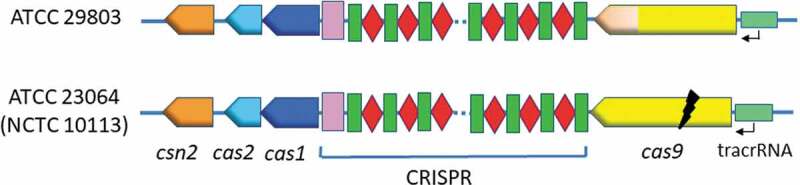
Schematic representation of the arrangement of CRISPR/Cas system components in two *M.salivarium* strains, ATCC 29803 and ATCC 23064 (NCTC 10113). The CRISPR array components are indicated as follows: red rhombus, spacer; green rectangle, direct repeat; pink rectangle, leader sequence. The black flash indicates a disruption in the gene sequence. ATCC, American Type Culture Collection; CRISPR, clustered regularly interspaced palindromic repeats; Cas, CRISPR-associated.

The CRISPR/Cas system of ATCC 29803 included the following components: the *csn2, cas2*, and *cas1* genes; the CRISPR array; the *cas9* gene; and the tracrRNA, in this order (Figure 5). This arrangement of the components was identical to that of ATCC 23064 (Figure 5).

### Identification of the rnc gene in ATCC 29803

The *rnc* gene was amplified in all five strains, yielding products of approximately 400 bp in each case (Figure 2(d)). The sequence of the PCR products of ATCC 29803 (Supplementary data 3) showed 90% similarity to that of ATCC 23064. *De novo* genome sequencing identified highly similar *rnc* gene sequences in ATCC 14277, 23557, and 33130.

## Discussion

This study investigated the presence and the sequences of the CRISPR/Cas system in four *M. salivarium* strains: ATCC 14277, 23557, 29803, and 33130. ATCC 23064 was used as a control for PCR amplification and sequencing.

Since PCR amplification could be done in ATCC 23064 and 29803, PCR amplicon sequencing via primer walking with paired-end reading was performed in these strains. The sequence of the CRISPR array of ATCC 23064 obtained using this approach was identical to that available on the NCBI database. Therefore, PCR amplification and sequencing via primer walking yielded sequences with high accuracy. As the CRISPR/Cas system sequences of ATCC 14277, 23557, and 33130 were not amplified via PCR, we performed *de novo* genome sequencing to search for the CRISPR/Cas system. However, no CRISPR/Cas systems were identified in these strains.

To detect and determine the sequence of the CRISPR/Cas system in this study, we performed PCR amplification and sequencing by primer walking as the initial steps, since primer walking was considered to have a higher accuracy than *de novo* genome sequencing. However, since whole-genome sequencing is widely being used due to its high accuracy and speed and reduced cost, owing to the advances in the analysis of genome sequences via next-generation sequencing [28], next-generation whole-genome sequencing can be applied to search for CRISPR/Cas systems in *Mycoplasma* and determine their genomic sequences.

In ATCC 29803, the CRISPR/Cas system comprises the *csn2, cas2*, and *cas1* genes, CRISPR array, *cas9* gene, and tracrRNA, in this order. Although the *csn2* gene was not annotated in the genome sequence of ATCC 23064, it was designated as *csn2* in this study, according to the annotation published by 29. The arrangement of these components was identical to that of ATCC 23064. Therefore, the CRISPR/Cas systems of ATCC 23064 and ATCC 29803 were classified as type II-A systems based on the presence of the *cas9, cas1, cas2*, and *csn2* genes.

CRISPR/Cas systems containing *cas9*, tracrRNA, *cas1, cas2*, and a CRISPR array, in this order, are the most common type found in the complete or draft genomes of *Mollicutes* [10]. However, inversions have been observed in the sequences of several strains, such as *M. dispar* ATCC 27140, *M. ovipneumoniae* NM 2010, *M. hyosynoviae* 232, *M. arginini* HAZ 145_1, and *Mycoplasma arthritidis* 158L3_1 [10]. In *M. hyosynoviae* 232 and *M. arthritidis* 158L3_1, the CRISPR arrays are located between the *cas9* and *csn2* gene loci [10]. No *Mycoplasma* species have been reported to contain the CRISPR/Cas system components in the same order as that of ATCC 23064 and ATCC 29803; however, the implications of this observation are unclear.

The number of DRs has been reported to range from 3–105 in 12 spp. of *Mycoplasma* studied [10]. Both ATCC 23064 and ATCC 29803 possessed 28 DRs. However, the length of DRs has been found to be 36 bp in 12 *Mycoplasma* spp [10]. In ATCC 23064 and ATCC 29803, all DRs were also 36 bp in length, except for one DR in ATCC 29803 that was shorter. This short DR was the 26^th^ DR in a series of 28 DRs. Small differences have been reported to occur; in particular, a repeat at the end of the CRISPR is often truncated or deviates from the consensus sequence [2].

The consensus sequences of all DRs were identical in ATCC 23064, although certain variants were observed in ATCC 29803. However, DR consensus sequences are relatively well-conserved in *Mycoplasma* species; the following underlined motifs: GTTTTAGCGCTGTACAATATTTGAGTAAGCTATAAC are conserved in most *Mycoplasma* spp [10]. These motifs were also found in the DR consensus sequences of ATCC 23064 and ATCC 29803.

The spacer sequences were unique to each strain and differed between the two strains. The target sequences, which bind to the crRNA-targeting sequences via complementary RNA–DNA base pairing, are different in every spacer when the CRISPR/Cas system functions as an endonuclease. Although a majority of the spacers are believed to be derived from phages, plasmids, or other organisms, certain CRISPR/Cas systems reportedly possess spacers matching sequences obtained from within the same genome and are called self-targeting spacers [30]. In ATCC 29803, 30 bp sequences corresponding to protospacers were not identified in the genomes of phages, plasmids, or viruses. Instead, certain homologs of spacer sequences were found in the coding sequences of *M. salivarium* strains ATCC 23064, 14277, 23557, and 33130. However, since the whole-genome sequence of ATCC 29803 was not analyzed, it was not certain whether these homologs were present in its own genome sequence.

It is not rare for a CRISPR-harboring bacterium to possess self-targeting sequences [30]. Although several hypotheses concerning the incorporation of self-targeting spacers and overcoming self-targeting by the CRISPR/Cas system have been reported [30], the mechanisms via which these spacers are acquired and integrated into the genome sequence and how the CRISPR-bearing bacteria survive self-targeting by their own CRISPR/Cas systems are unknown.

For ATCC 23064, the *cas9* gene sequence is disrupted in the middle by a frameshift mutation generated by a UAA stop codon. ATCC 29803 had no stop codon within the *cas9* gene sequence and the Cas9 protein of ATCC 29803 was predicted to contain 1,203 amino acids. The Cas9 proteins present in *Mycoplasma* spp. are larger than *S. pyogenes* Cas9 (1,059 amino acids), but smaller than *Staphylococcus aureus* Cas9 (1,368 amino acids), with sizes ranging from 1,069 to 1,272 amino acids [10]. The size of the Cas9 protein of ATCC 29803 was within this range, which is considered large enough to be a type-II crRNA-guided endonuclease.

The *cas9* gene sequence of ATCC 29803 showed 98% identity to that of ATCC 23064. However, approximately 500 bp of sequence on the CRISPR array side showed no significant similarity to that of ATCC 23064. The Cas9 protein consists of a recognition lobe and a nuclease lobe, which includes several domains. The HNH and RuvC nuclease domains, which are contained in the nuclease lobe, are conserved, whereas the protospacer adjacent motif (PAM)-interacting (PI) domain is variable [31]. Approximately 500 bp of the *cas9* gene sequence on the CRISPR array side in ATCC 29803, which was different from that of ATCC 23064, may include the PI domain encoding region. Therefore, when the *cas9* gene of ATCC 23064 is not disrupted and coded, Cas9 functions as an endonuclease, and recognizable PAM sequences may differ between the two strains.

It is reported that the Cas9 protein in *Mycoplasma* spp. is structurally related to the *S. aureus* Cas9 except for the PI domain, suggesting that various PAMs may be recognized by Cas9 of different *Mycoplasma* spp [10]. The difference in the sequence of the PI domain between ATCC 23064 and ATCC 29803 may suggest that PAM sequences vary among *M. salivarium* strains.

The structure of the predicted crRNA/tracrRNA hybrid is conserved and shows typical stem-loop structures among the *Mycoplasma* spp [10]. Since the tracrRNA sequences were identical between ATCC 23064 and ATCC 29803, the secondary structures of the predicted crRNA/tracrRNA hybrids formed two stem loops, including a nexus stem-loop and a terminator ending, with a poly-uridine tract, in both strains. The *rnc* genes required by type-II CRISPR/Cas systems to process pre-crRNA to mature crRNA were identified in ATCC 29803 and the other strains. From the above findings, it can be inferred that ATCC 29803 possesses the genomic components necessary to express the type-II CRISPR/Cas system, which potentially functions as an RNA-guided endonuclease.

We restricted this study to the screening of the CRISPR/Cas system and the determination of its sequence in four ATCC strains of *M. salivarium*. Therefore, the function of the CRISPR/Cas system in ATCC 29803 as an RNA-guided endonuclease was not evaluated.

In conclusion, this study showed that only ATCC 29803 possessed a genomic sequence of the type II-A CRISPR/Cas system among the four *M. salivarium* strains ATCC 14277, 23557, 29803, and 33130. By comparing the sequences of the CRISPR/Cas systems between ATCC 29803 and ATCC 23064, we found that the sequences were almost similar except for the spacer sequences and regions in the *cas9* gene sequence. The spacer sequences are unique to each strain and differed between the two strains. The *cas9* gene of ATCC 23064 was disrupted by a UAA stop codon, whereas that of ATCC 29803 lacked a stop codon. The *cas9* gene sequence of ATCC 29803 showed 98% identity to that of ATCC 23064. However, approximately 500 bp of the sequence on the CRISPR array side, which contained the PI domain encoding region, showed no similarity to that of ATCC 23064.

## Supplementary Material

Supplemental Material

## Data Availability

Complete genome sequence data of NCTC 10113 is available in the NCBI database under the accession number: NZ_LR214939. Nucleotide sequencing data of the CRISPR region in ATCC 23064 is available in the DDBJ database under the accession number: LC633536. Genomic sequencing data of the type-II CRISPR/Cas system of ATCC 29803 is available in the DDBJ database under the accession number: LC628936. De novo whole genome shotgun sequencing data of ATCC 14277 is available in the DDBJ database under the accession numbers: BPLV01000001–01000007. De novo whole genome shotgun sequencing data of ATCC 23557 is available in the DDBJ database under the accession numbers: BPLW01000001–01000006. De novo whole genome shotgun sequencing data of ATCC 33130 is available in the DDBJ database under the accession numbers: BPLX01000001–01000009.
