# Supplementary material for: A genomic sequence of the type II-A clustered regularly interspaced short palindromic repeats (CRISPR)/CRISPR-associated system in Mycoplasma salivarium strain ATCC 29803
Source: J Oral Microbiol. 2022 Jan 2;14(1):2008153. doi: 10.1080/20002297.2021.2008153 (PMC8725752; doi:10.1080/20002297.2021.2008153)
Supplement: Supplemental Material [file ZJOM_A_2008153_SM0070.docx]

Supplementary Material

# Supplementary Data

Supplementary Data 1. Analysis of sequence similarity of the *cas9* gene between *Mycoplasma salivarium* strain ATCC 23064 (NCTC 10113) and *M*. *salivarium* strain ATCC 29803, using a multiple sequence alignment program, Clustal Omega. Asterisks indicate that two nucleotides are identical in a pair of that of two strains.

ATCC23064 ATGAAAGAAAAAAAGAATGTTATATTAGGTTTTGACTTAGGTGTTGGTTCTGTCGGTTGA 60

ATCC29803 ATGAAAGAAAAAAAGAATGTTATATTAGGTTTTGACTTAGGCGTTGGTTCTGTCGGCTGA 60

***************************************** ************** ***

ATCC23064 GCTATTGTTGATAGTGAAACAAATGATATTATTAAGTTAGGTTCTAGATTATTTAACGAA 120

ATCC29803 GCTATTGTTGATAGTGAAACAAATGATATTATTAAGTTAGGTTCTAGATTATTTAACGAA 120

************************************************************

ATCC23064 CCTGAACTTGCTGCAGATCGAAGAGGTTTTCGGGGAATTCGTAGATTAATAAGACGTAGA 180

ATCC29803 CCTGAACTTGCTGCAGATCGAAGAGGTTTTCGGGGAATTCGTAGATTAATAAGACGTAGA 180

************************************************************

ATCC23064 CAATATAGAAATGATAAATTTTATCGTTTAATTTTAAAATATAAGGATATATTCGGTTTT 240

ATCC29803 CAATATAGAAATGATAAATTTTATCGTTTAATTTTAAAATACAAGAATATATTCGGTTTT 240

***************************************** *** **************

ATCC23064 GGAAATCGCAAGGATATTGAACAAATGTTTGTTAAGTTGAATAGGAAATATTCTAACATT 300

ATCC29803 GGAAATCGTAAGGATATTGAACAAATGTTTGTTAAGTTGAATAGGAAATATTCTAACATT 300

******** ***************************************************

ATCC23064 TTGGAGTTAAAGATTCTATCTTTAAATAAACAAATTAATGTTCAAGAATTGGTTTGACTG 360

ATCC29803 TTGGAGTTAAAGATTTTATCTTTAAATAAACAAATTAATGTTCAAGAATTGGCTTGACTG 360

*************** ************************************ *******

ATCC23064 TTGCATGATTACTTAGAAAATCGTGGGTATTTTTATGAAGATGTTGACAGCCTAGAAGAA 420

ATCC29803 TTGCATGATTACTTAGAAAATCGTGGGTATTTTTATGAAGATGTTGACAGCCTAGAAGAA 420

************************************************************

ATCC23064 ATTGAAAAAAATGATGTTAAAACAACATTATTTCCTTCACAAATACTGTTTAATTTCTAT 480

ATCC29803 ATTGAAAAAAATGATGTTAAAACAACGTTATTTCCTTCACAAATACTGTTTAATTTTTAT 480

************************** ***************************** ***

ATCC23064 AAAAAATATGGAACAGCTAAATCATTTAATAGCTATATAGATGATGAATTTAGATACAAT 540

ATCC29803 AAAAAATATGGAACAGCTAAATCATTTAATAGCTATATAGATGATGAATTTAGATACAAT 540

************************************************************

ATCC23064 TTTTCAAATAAAAATTGATTGCATGAACTAAATAAATTATTTGAAATTCAAAAAACTGAT 600

ATCC29803 TTTTCAAATAAAAATTGATTGCATGAACTAAATAAATTATTTGAGATTCAAAAAACTGAT 600

******************************************** ***************

ATCC23064 GCAAATTTTATAAATGATTATTTAAAGATATTTACTGCTATAAGACCATATGCTAAAGGG 660

ATCC29803 GCAAATTTTATAAATGATTATTTAAAGATATTTACTGCTATAAGACCATATGCTAAAGGG 660

************************************************************

ATCC23064 CCTGGCAGTATAAATAGTTATTCTGAATATGGAAGATTTAAATATAAAGATAATAAACTT 720

ATCC29803 CCTGGCAGTATAAATAGTTATTCTGAATATGGAAGATTTAAATATAAAGATAATAAACTT 720

************************************************************

ATCC23064 GTCGAAGCATATAGCAATATTTGGGAGAAAACTATTGGAAAATGTAGTATTTTTCCATCT 780

ATCC29803 GTCGAAGCATATAGCAATATTTGGGAGAAAACTATTGGAAAATGTAGTATTTTTCCATCT 780

************************************************************

ATCC23064 GAATTAAGAGCGGTAAAGAATTGTGCTAGTCATGAAATTTTTAATATTTTAAATGAACTT 840

ATCC29803 GAATTAAGAGCGGTGAAGAATTGTGCAAGTCATGAAATTTTTAATATTTTAAATGAACTT 840

************** *********** *********************************

ATCC23064 AATAATTTAAAAAATAGCGAATATTTAGATTGAAGATTAACAAAAGAAAATAAATTAGTA 900

ATCC29803 AATAATTTAAAAAATAGCAAATATTTAGACTGAAGATTAACAAAAGAAAATAAATTAGTA 900

****************** ********** ******************************

ATCC23064 ATTTTGAATGATTTGCTAAATGAATTTTCAACATCAAAAAGTTACAAAGGTTTAAAAGCA 960

ATCC29803 ATTTTGAATGATTTGCTAAATGAATTTTCAACATCAAACAGTTACAAAGGTTTAAAAACA 960

************************************** ****************** **

ATCC23064 AAGACTTTTATTCCATTTTTAGAAACTTATTGTTTAAATAATAATTTGCCCTTTTCATTA 1020

ATCC29803 AAAACTTTTATTCCATTTTTAGAAACTTATTGTTTAAATAATAATTTGCCCTTTTCATTA 1020

** *********************************************************

ATCC23064 GATGAATTGAATAAGATTGATAATCAAGGATTTAATGGATTTAAATACGATAAAGAGACT 1080

ATCC29803 GATGAATTGAATGAGATTGATAATCAAGGATTTAATGGATTTAAATACAATAAAGAGACT 1080

************ *********************************** ***********

ATCC23064 ACAAAACCTGAGTTTGTTGAAATTAGAAATATTTTTGAAATTATTAGAGTTTTGGCGGAA 1140

ATCC29803 ACAAAACCTGAGTTTGTTGAAATTAGAAATATTTTTGAAATTATTAGAGTTTTGGTGGAA 1140

******************************************************* ****

ATCC23064 TTTGGTATTAATACAAATGTATTAACCTTTAATGATGATTTAGTAAAACAATATTATCAA 1200

ATCC29803 TTTGGCATTAATACAAATGTATTAACCTTTAATGATGATTTAGTAAAACAATATTATCAA 1200

***** ******************************************************

ATCC23064 TCAGATTTAAAATATGAACCCACAACTTGAATAACCTTATTTAATAGAATTGTAGATACT 1260

ATCC29803 TCAGATTTAAAATATGAACCCACAACTTGAATAACCTTATTTAATAGAATTGTAGATACT 1260

************************************************************

ATCC23064 TTGAATAATTTCAAAGACAAAGAAAATAGAATTGAAAAATTAAATATTCTTAAAGAATTG 1320

ATCC29803 TTGAATAATTTCAAAGACAAAGAAAATAGAATTGAAAAATTAAATATTCTTAGAGAATTG 1320

**************************************************** *******

ATCC23064 TTTGAATCAATTTTTAATGATATTAAAGATGAGAAACTTGCAAATATTATTGAAGCCATT 1380

ATCC29803 TTTAAATCAATTTTTAATGATATTAAAGATGAGAAACTTGCAAATATTATTGAAGCCATT 1380

*** ********************************************************

ATCC23064 GCAATTAACAAGAAAATTAAGGCAAGTACTACTGCATCTTTATCATTAAAAGCTTTAAAT 1440

ATCC29803 GCAATTAACAAGAAAATTAAGTCAAGTACTACTGCATCTTTATCATTAAAAGCTTTAAAT 1440

********************* **************************************

ATCC23064 TTGTTTTGACCAGAAATGTTAAATACTAATTCAAATTTTGAACAATTGAAATTTAATCCA 1500

ATCC29803 TTGTTTTGACCAGAAATGTTAAATACTAATTCAAATTTTGAACAATTGAAATTTAATCCA 1500

************************************************************

ATCC23064 AGTTTTTTTTAATAAAGAAGATTACAAAGATAATTTTGAGCAAACTGGTAAGTATATTAA 1560

ATCC29803 AGTTTT-TTTAATAAAGAAGATTACAAAGATAATTTTGAGCAAACTGGTAAGTATATTAA 1559

****** *****************************************************

ATCC23064 TTTAAAAAGTTTAGATAATCAAATTATTCCTCCATCAGTTAAAGCTACAATAAGAGAAGC 1620

ATCC29803 TTTAAAAAGTTTAGATAATCAAATTATTCCTCCATCAGTTAAAGCTACAATAAGAGAAGC 1619

************************************************************

ATCC23064 TATTAGCATTTTTAATAAAATTAAAAAATTATATTCAAATGAATTTAATATTACAAAAGT 1680

ATCC29803 TATTAGCATTTTTAATAAAATTAAAAAATTATATTCAAATGAATTTAATATTACAAAAGT 1679

************************************************************

ATCC23064 TGTTGTTGAATTGGCACGTGAAAAGAATGATAAAGAACAACGTGATACTATTACTAAAAT 1740

ATCC29803 TGTTGTTGAATTGGCACGTGAAAAGAATGATAAAGAACAACGTGATACTATTACTAAAAT 1739

************************************************************

ATCC23064 TAATAATATAAATAAGAAAAGAAAAGAAGATGCTATTAAATTAGTAAATGATTTAGAACC 1800

ATCC29803 TAATAATATAAATAAGAAAAGAAAAGAAGATGCTATTAAATTAGTAAATGATTTAGTACC 1799

******************************************************** ***

ATCC23064 AAATTTATTTGATAAAGAATCAATTGAAAAGAAAGCATTTAAAGTTTTTTTATATCAACA 1860

ATCC29803 AAATTTATTTGATAAAGAATCAATTGAAAAGAAAGCATTTAAAGTTTTTTTATATCAACA 1859

************************************************************

ATCC23064 ACAAGATGGAAGAGATCCATATTCAGGTCAATTTATGAATTTACCTGAACTAATAAAAAA 1920

ATCC29803 ACAAGATGGATGAGATCCATATTCAGGCCAATTTATGAATTTACCTGAACTAATAAAAAA 1919

********** **************** ********************************

ATCC23064 TGATAACTATTGTGAAATTGATCACATTCTACCCTATTCCAAATCTGCAAATGATTCTAT 1980

ATCC29803 TGATAACTATTGTGAAATCGATCACATTCTGCCCTATTCCAAATCTGCAAATGATTCTAT 1979

****************** *********** *****************************

ATCC23064 TTCAAATAAAGTTTTGGTTTTAAAATCTAGCAATCAAATTAAAGGAAATAGAATTCCATA 2040

ATCC29803 TTCAAATAAAGTTTTGGTTTTAAAATCTAGCAATCAAATTAAAGGCAATAGAATTCCATA 2039

********************************************* **************

ATCC23064 TGATTATTTCCAAAATACTGTTTTAGAAAATGGTTGAAATTGACAAGAATATAAAAATTG 2100

ATCC29803 TGATTATTTTCAAAATACAGTTTTAGAAAATGGTTGAAATTGACAAGAATATAAAAATTG 2099

********* ******** *****************************************

ATCC23064 AGCTTATAAAGTTATTTTAAATGGTGACTTAACCAAATTTTCAAACAAAAATCAAAGAAT 2160

ATCC29803 AGCTTATAAAGTCATTTTAAATGGCGACTTAGCCAAATTTTCAAACAAAAATCAAAGAAT 2159

************ *********** ****** ****************************

ATCC23064 ACAAAAATACAATAATTTAGTTAAAGAAAAATTTGACGAATATGATCAATTAGATTTTTT 2220

ATCC29803 ACAAAAATACAATAATTTAGTTAAAGAAAAATTTGACGAATATGATCAATTAGATTTTTT 2219

************************************************************

ATCC23064 AAGTAGAAATTTAAATGATACAAGATATGCTACAAAATATTTTCGTGACTTACTAGGGAA 2280

ATCC29803 AAGTAGAAATTTAAATGATACAAGATATGCTACAAAATATTTCCGTGACTTACTAGGAAA 2279

****************************************** ************** **

ATCC23064 TTATTCAAAAAATCATAATGATGAATTTCGTGTTATTTGTATGAATGGTAGTGTAACATC 2340

ATCC29803 TTATTCAAAAAATCATAATGATGAATTTTGTGTTATTTGTATGAATGGTAGTGTAACATC 2339

**************************** *******************************

ATCC23064 ATATTTTAGAAAAAAAGCTTGTTTAATCAAAGACAGAGATGATTATTCTCATCATGCACA 2400

ATCC29803 ATATTTTAGAAAAAAATCTTGTTTAATCAAAGACAGAGATGATTATTCTCATCATGCGCA 2399

**************** **************************************** **

ATCC23064 AGATGCTTCTATTATTGCGATTATTGCCAATAAATCAAAAAGTATTTTTAATTTGTTATT 2460

ATCC29803 AGATGCTTCTATTATTGCAATTATTGCCAATAAATCAAAAAGTATTTTTAATTTGTTATT 2459

****************** *****************************************

ATCC23064 ACATGATGATAGACAAATTAGCTATATGTCTAAAGACGGAACCATAAGACTAATGGATAA 2520

ATCC29803 ACATGATGATAGACAAATTAACTATATGTCTAAAGACGGAACCATAAGACTAATGGATAA 2519

******************** ***************************************

ATCC23064 AGAAACTGGAGAAATTATTGAAGAAGATTTATCTCCAGAAGATCCTAGATTTAATAAAAC 2580

ATCC29803 AGAAACTGGAGAAATTATTGAAGAAGATTTATCTCCAGAAGACCCTAGATTTAATAAAAC 2579

****************************************** *****************

ATCC23064 TGATTTAGCTATTCAAGATATTGCAGAAACAATTAATGAAAAAATTGCTAATACTATTGG 2640

ATCC29803 TGATTTAGCTATTCAAGATATTGCAGAAACAATTAATGAAAAAATTGCTAATACTATTGG 2639

************************************************************

ATCC23064 CGATGTTATGTTTTCTAGAAAAACGGTTATAAAAACTAATCCTTCTATTTCAGATCAAAC 2700

ATCC29803 CGATGTTATGTTTTCTAGAAAAACGGTTATAAAAACTAATCCTTCTATTTCAGATCAAAC 2699

************************************************************

ATCC23064 AATTTATGGGTATAGAAAAATTAGTGAAGATAGTGATGAAATATTGCAAATTCAAAAATT 2760

ATCC29803 AATTTATGGGTATAGAAAAATCAGTGAAGATAGTGATGAAATATTACAAATTCAAAAATT 2759

********************* *********************** **************

ATCC23064 AAATTTATTTGAAGTTGATGCAAAAAACAAGAAGTCTAAAGAAAATGTAAAATTGTCTGA 2820

ATCC29803 AAATTTATTTGAAGTTGATACAAAAAACAAGAAGTTTAAAGAAAATGCAAAATTGTTAGA 2819

******************* *************** *********** ******** **

ATCC23064 TTTTTTTGGCGAGAATCCGAAACTTAGAGAAAATTTGTTAATTTATAAGTCTCATAAATC 2880

ATCC29803 TTTCTTTGGTGAGAATCCAAAGCTTAGAGAAAACTTGTTAATTTATAAGTCTCATAAGTC 2879

*** ***** ******** ** *********** *********************** **

ATCC23064 AGAATATGAAAAACTAAATAATATTTATATGCAATATAAGGAAAAAAATGAAAAAGCTCC 2940

ATCC29803 AGAATATGAAAAGCTAAATAATATCTATATGCAATATAAGGAAAAAAATGAAAAAGCTCC 2939

************ *********** ***********************************

ATCC23064 ATTTACTGCATATATGAAGGATTTAACAAATATTGCTCCAGAAATATTCACTACTAACTT 3000

ATCC29803 ATTTACTGCATATATGAAGGATTTAACAAATATTGCTCCAGAAATATTTACTGCTAACTT 2999

************************************************ *** *******

ATCC23064 AATTAACAACTATATGTCAAATGGTAAGGTAGTTGTGTTTGATCCCACTTCTAAAAAACA 3060

ATCC29803 AATTAACAACTATATGTCGAATGGTAAGGTAGTTGTGTTTGATCCAACTTCTAAAAAACA 3059

****************** ************************** **************

ATCC23064 AACATTTGTTAAACATTTAAAGTATTTTTTTCCTGACAAAAAAGATTTAAATGTTGTTTT 3120

ATCC29803 AACATTTGTTAAACATTTAAAGTATTTTTTTCCTAAAACAAAAAATATGCAAAATGTTCT 3119

********************************** * * **** ** * * **** *

ATCC23064 ACTAAACAAAAAACAAAATAATAAATCGTTTGTGGAAAATTTAAAATCAATTGGTGTTCT 3180

ATCC29803 TTTAAACAAAAAACAAAAAAATAAATCATTCTATGAGAATTTGAATTCAATTGGTGCATT 3179

**************** ******** ** ** ***** ** ********** *

ATCC23064 TGTTTACAAAAACAAAAAAGATAAATATGAAATTATTGGAATTAATGCTTTGTTGTATAA 3240

ATCC29803 AGTTTATATCAATAAGAAAGGAACATATGATTTTATTGGAATAAATGCATTGTTACTTAA 3239

***** * ** ** **** * ****** ********** ***** ***** ***

ATCC23064 ATTTAATAATAATGTTAAAAAAAATAAATTATTAATTGAAAATAATTATGACAATTCAAT 3300

ATCC29803 ATTTAATATGAAAAACGGTGTCATTAATTATTTAGACGAGAATATTTATGATTTAGCAAC 3299

******** ** * *** * *** ** **** ****** ***

ATCC23064 ATTACAGAAGGTTAAAAATAATAAAAATATTCCAATAGATAATAAACCAATTAGTATTTT 3360

ATCC29803 TATAAATGCTTATAAAGTTTTAAAAAATATTGATGTTTCATCTAAACCCGTTCATTATTT 3359

** * **** * ********* * ****** ** * ***

ATCC23064 ATTTTCTAATGGAACTTTATTAAAAAATAAATTGACTAATCAAATTTTTATAGTAACAAG 3420

ATCC29803 GTTTGTTAATGGGACTATTCTTCAAAAGAAATCTGATAATAATTTGTTTATTGTTGTTGG 3419

*** ****** *** * * **** **** **** * * ***** ** *

ATCC23064 TTTTACACCATCACTTTCCACTATAGAAATAAAACCGATTTTTATGTCAACCACGAAATA 3480

ATCC29803 TATGGTTCCTTCGAATGGAACTATTGAATTGAAATGCATCTCTATGTCAAATGCAAATTA 3479

* * ** ** * ***** *** * *** ** * ******** * ** **

ATCC23064 TCTAAAAATAATTAAAAATAAAAA------AAGTGCAACAACCAGAATGATATTTACTAT 3534

ATCC29803 TTTTTCAATTCCTGCTGATGAAAATGGGAAAAGGCCAATAAAAAGAATTAGACTATCTAT 3539

* * *** * ** **** *** *** ** ***** * * * ****

ATCC23064 AAATTCTTTATTTGATAAATTTGAAATAGTTAATTTAGATGTTATTGGTAATCTTTTTGA 3594

ATCC29803 CAATAGTATAATGAATGAATATATTGTTTGTAGTGTTGACGTTTTGGGAAACATATACAA 3599

*** * ** * ** *** * * ** * * ** *** * ** ** * * *

ATCC23064 GTAG------------ 3598

ATCC29803 GCCGCAGAATTAATAA 3615

* *

**Supplementary Data 2.** Theoretical Cas9 protein sequence (1203 amino acids) of *Mycoplasma salivarium* strain ATCC 29803, translated using ExPASy based on the *cas9* gene sequence

MKEKKNVILGFDLGVGSVGWAIVDSETNDIIKLGSRLFNEPELAADRRGFRGIRRLIRRRQYRNDKFYRLILKYKNIFGFGNRKDIEQMFVKLNRKYSNILELKILSLNKQINVQELAWLLHDYLENRGYFYEDVDSLEEIEKNDVKTTLFPSQILFNFYKKYGTAKSFNSYIDDEFRYNFSNKNWLHELNKLFEIQKTDANFINDYLKIFTAIRPYAKGPGSINSYSEYGRFKYKDNKLVEAYSNIWEKTIGKCSIFPSELRAVKNCASHEIFNILNELNNLKNSKYLDWRLTKENKLVILNDLLNEFSTSNSYKGLKTKTFIPFLETYCLNNNLPFSLDELNEIDNQGFNGFKYNKETTKPEFVEIRNIFEIIRVLVEFGINTNVLTFNDDLVKQYYQSDLKYEPTTWITLFNRIVDTLNNFKDKENRIEKLNILRELFKSIFNDIKDEKLANIIEAIAINKKIKSSTTASLSLKALNLFWPEMLNTNSNFEQLKFNPSFFNKEDYKDNFEQTGKYINLKSLDNQIIPPSVKATIREAISIFNKIKKLYSNEFNITKVVVELAREKNDKEQRDTITKINNINKKRKEDAIKLVNDLVPNLFDKESIEKKAFKVFLYQQQDGWDPYSGQFMNLPELIKNDNYCEIDHILPYSKSANDSISNKVLVLKSSNQIKGNRIPYDYFQNTVLENGWNWQEYKNWAYKVILNGDLAKFSNKNQRIQKYNNLVKEKFDEYDQLDFLSRNLNDTRYATKYFRDLLGNYSKNHNDEFCVICMNGSVTSYFRKKSCLIKDRDDYSHHAQDASIIAIIANKSKSIFNLLLHDDRQINYMSKDGTIRLMDKETGEIIEEDLSPEDPRFNKTDLAIQDIAETINEKIANTIGDVMFSRKTVIKTNPSISDQTIYGYRKISEDSDEILQIQKLNLFEVDTKNKKFKENAKLLDFFGENPKLRENLLIYKSHKSEYEKLNNIYMQYKEKNEKAPFTAYMKDLTNIAPEIFTANLINNYMSNGKVVVFDPTSKKQTFVKHLKYFFPKTKNMQNVLLNKKQKNKSFYENLNSIGALVYINKKGTYDFIGINALLLKFNMKNGVINYLDENIYDLATINAYKVLKNIDVSSKPVHYLFVNGTILQKKSDNNLFIVVGMVPSNGTIELKCISMSNANYFSIPADENGKRPIKRIRLSINSIMNEYIVCSVDVLGNIYKPQN

**Supplementary Data 3.** Partial *rnc* gene sequence of *Mycoplasma salivarium* strain ATCC 29803

agcagtacatcaaacgttatgatctattgctaacaatttacactttcctaattatgtacgtgcttgaaaaatgcttttgcaatggtaaaaatgataaagtgaattcagatttctatgaagcatttacaggagctatttattttgaaaaaggccctgagttttttaccaaatttttgaaacatgacttagctacttatattcctaatgtgactcacgatgatttaattgatgcaaatctgagtttcagaagttattcaaatgcaaggaattagtaaaataatttatgaatcaaaacctatgaatgaaaaaaaattttcacgtagcgttattgggacccacatgaaag
